# Development and Validation of the Chinese Modesty Scale (CMS)

**DOI:** 10.3389/fpsyg.2018.02014

**Published:** 2018-10-23

**Authors:** Mimi Xiong, Fengyan Wang, Ruixue Cai

**Affiliations:** ^1^Institute of Moral Education, Nanjing Normal University, Nanjing, China; ^2^School of Psychology, Nanjing Normal University, Nanjing, China; ^3^School of Public Administration, Nanjing Normal University, Nanjing, China

**Keywords:** value modesty, instrumental modesty, modesty, scale development, Chinese

## Abstract

This paper describes the development and method of validation of the Chinese Modesty Scale (CMS). Based on Wang’s dualistic model for value and instrumental modesty, our study employed a review of the literature, in-depth interviews, open-ended investigations, and feedback from experts. An initial 14-item scale for analyzing the issue of “Chinese modesty” was developed. Then we explored the dimensions and final items of this CMS using item analysis and exploratory factor analysis (EFA) with sample 1 (*n* = 406). After that, we conducted a confirmatory factor analysis (CFA) to replicate the factor structure obtained through EFA with a refined, independent, 12-item scale (*n* = 662). Results confirmed the dualistic model (for value and instrumental modesty) on which this scale was based. That is, we found that there are two kinds of “Chinese modesty”: value modesty and instrumental modesty. As a valid, reliable scale, the CMS can therefore be used to measure the “Chinese modesty” of/in different age groups.

## Introduction

In the book *I Ching* [The book of change], an ancient Chinese divination book and one of the oldest of the Chinese classics, it said “One who keeps modest all the time so as to cultivate his/her moral character is a gentleman” (Huang and Zhang, 2016). In China, most people regard modesty as a good moral attitude and only arrogant people enjoy boasting in front of others (Wang and Zheng, 2015). Chinese people who have positive self-concept and interpersonal skills value modesty highly and get used to showing their talents moderately in order not to offend others and gain social approval (Yang, 1996). Lao Tzu, one of the most famous Chinese philosophers, also said, “The great rivers and seas get their kingship over the hundred lesser streams through the merit of being lower than they” (Chen, 2009). In China, such sayings have become commonly accepted truths about modesty, with Chinese children as young as nine having been socialized to behave modesty in public (Lee et al., 1997; Fu et al., 2010). For Chinese people, modesty is not only an understanding of real life but also a moral demand for ideal personality characteristics (Miao and Liang, 2012).

Previous scholars mainly defined modesty from two perspectives. First, they regarded modesty as a stable personality characteristic. The six-factor personality model (HEXACO) proposed by Lee and Ashton (2004) included an important factor named Honesty-Humility. Chinese humility, as found in later studies, includes four dimensions: modesty, pompous-avoidance, arrogant-avoidance, and vanity-avoidance (Yang et al., 2015). Yan (2010) argued that modesty is a kind of personality characteristic derived from moral traits and real modesty should include honesty, aggressiveness, stability, discontent, lack of pride, and lack of stereotyping. Second, scholars have regarded modesty as one’s cognitive and behavioral tendencies. Zhu xi, a famous Neo-Confucianism in the Southern Song Dynasty, said in his book *Zhuzi Yulei* [Quotations from zhuxi], “Ordinary people see only their own advantages and others’ disadvantages. However, modest people, more than this, choose to lower themselves to treat others on an equal footing” (Li, 1986). Obviously, Zhu Xi believed that modesty is the integration of belittling ourselves and raising others. These two factors, one internal and one external, constitute the essence of modesty (Lyu, 2007). Driver (1999) held a similar viewpoint: a modest individual may underestimate or even ignore his/her self-worth. However, Emmons (2000) argued that humility is a kind of spiritual intelligence, and to be modest is not to have a low opinion of oneself; rather, it is to have a realistic appraisal of one’s strengths and weaknesses. Besides accurate self-perception, Tangney (2000) proposed that modest individuals also have these following five characteristics: (a) an ability to acknowledge their own mistakes, imperfections and limitations; (b) openness to new ideas and contradictory information; (c) rational views of others’ abilities and achievements; (d) an ability to forget the self; and (e) an appreciation of the value of all things. Kim et al. (2005) argued that humility is one of the most important components of Asian-American values. Hu (2007) argued that self-modesty is a harmony control process through unassuming self-presentation, firmness-restraining flexibility, and strength-defeating weakness, indicating that modest people’s self-deprecating behavior is essentially a kind of positive self-presentation and its purpose is to make progress through these superficial concessions. It’s necessary to mention that previous empirical studies have found that laypeople’s views of humility and modesty are highly similar (Elliott, 2010). For example, Exline and Geyer (2004) asked participants to rate the extent to which they perceived humility as similar to modesty on a scale ranging from 0 (*not at all*) to 10(*extremely*). The results showed that humility was seen as similar to modesty (*M* = 7.80) and almost half of participants used the word “modesty” in their definitions of humility. Similarly, Gregg et al. (2008) found humble was emerged as one of the central characteristics of modest people. In addition, some researchers considered modesty an important subdomain of humility (Lee and Ashton, 2004; Davis et al., 2011, 2016). Taken together, these above researches suggest that there is considerable overlap between modesty and humility, the present study thus don’t distinguish clearly between the two concepts.

Hu (2007) divided Chinese modesty into two types, real modesty and false modesty, with the degree of sincerity being the key difference between them. Real modesty is not only an accurate reflection of one’s moral cultivation but also an explicit indication of one’s life attitude that there will always exist room for improvement. It has two characteristics: sincerity and moderation. False modesty can be seen as a kind of self-presentation strategy with the aim of achieving other utilitarian purposes, and it has two characteristics: hypocrisy and cowardice. In addition, many researchers have categorized modesty as either situational or trait (Tangney, 2000; Cai et al., 2011). Situational modesty mainly describes people’s modesty behaviors in different situations, and it has been widely confirmed that modesty behaviors observed in different contexts are not the same (Watling and Banerjee, 2007; Fu et al., 2011; Heyman et al., 2011). For instance, Han (2012) found people like to take an immodest attribution style (attributing to ability, effort, etc.) when their achievements do not pose a threat to others and the relationship is close. In other cases, they tended to adopt a modest attribution style, attributing success to extrinsic factors (luck, task difficulty, etc.). Similarly, in public or in the presence of authorities, people like to keep a low profile and not show off their strengths or abilities. In this case, modesty can be considered as a kind of impression management tactic (Chen et al., 2009; Diekmann et al., 2015). Trait modesty refers to one’s general tendencies toward self-effacement, other enhancement, and avoidance of attention seeking (Chen et al., 2009). For example, Garcia (2006) divided trait modesty into inward-directed and outward-directed. The former is seen primarily as a matter of people’s spontaneous and stable tendencies toward their envied features; the latter refers to people’s evaluations, under the influence of others, of their own achievements.

There have been extensive investigations into the measurement of modesty using Westerners samples, such as Whetstone’s Modesty Response Scale (MRS; cited by Cialdini et al., 1998; Kurman, 2002), Humility Scale (HS; Elliott, 2010), and Relational Modesty Scale (RMS; Davis et al., 2016). These modesty scales have logical subscales and satisfactory psychometric properties. However, they also have two important shortcomings: (1) most of these modesty scales formed their dimensions by statistical methods, and thus lack a social, cultural and historical basis; (2) they lack cultural specificity. Modesty is highly valued both in the East and West (Elliott, 2010). Meanwhile, it has obvious cultural relativity (Lee et al., 1997; Fu et al., 2010; Heyman et al., 2011). However, these modesty scales were primarily developed and validated in Western cultural settings. Is Chinese modesty the same as Western modesty? There are no answers in these scales. As for measurement in-equivalences between Chinese and Western modesty, a recent survey conducted by McGrath (2015) provided a direct evidence. The Values in Action-Inventory of Strengths (VIA-IS), which includes a subscale on Humility/Modesty, was conducted by McGrath in a sample of 15,540 individuals from 16 nations (six Eastern countries included) to examine its measurement invariances. Results showed that most of VIA-IS subscales have achieved configural and metric invariance, but not for humility/modesty subscale. Consequently, it seems that direct comparison of scores in humility/modesty subscale is not reasonable and this result can lend support to the cross-cultural differences of the modesty.

Based on a review of the existing literature, we consider that there are at least five significant differences in modesty between Westerner and Chinese: (1) the differences in the origins of modesty between China and the West. Chinese’s modesty comes mainly from one’s moral self-cultivation. The reason for this is that, in China, people’s thoughts and behaviors are deeply shaped by Confucianism, Taoism, and Buddhism, and all these doctrines were full of praise for modesty (Li, 1986; Keown, 1992; Chen et al., 2009). Westerner’s emphasis on modesty is mainly influenced by Christian doctrine (Tangney, 2000; Elliott, 2010). Specifically, in Western countries, humility is regarded as one of the seven heavenly virtues (Thomson, 1949) and humility express the subordination of the human being to lord God (Dyson, 2006); (2) Chinese and Westerner have different degrees of modesty. Chinese are often overly modest and give a self-repression impression. Different from this, Westerners know when enough is enough and generally do not use modesty to repress themselves; (3) contemporary Westerner and Chinese have different attitudes toward modesty. Modesty has been regarded as virtue in traditional Western and Chinese culture. But in modern culture, comparing with Chinese, Westerners value personal achievement (Markus and Kitayama, 1991; Rui and Stefanone, 2013), self-uniqueness (Yokota, 2012), and positive self-presentation (Lee-Won et al., 2014) more highly, and thus, modesty is less encouraged (Chelminski and Coulter, 2006); (4) Chinese and Westerner have different preferences for modesty: Chinese prefer value modesty and Westerners, in order to maximize their own interests, are more concerned with instrumental modesty; (5) Chinese and Westerner have different underlying purpose of being modest. Chinese often adopt instrumental modesty to protect themselves from the consequences of the envies of mean man. However, in modern Western culture, the notion of equality, competition and justice are deeply rooted, and positive self-presentation has gained wider recognition. Hence, there is no need for Westerners to hide talents to avoid other’s envy. These five differences were proposed based on systematic literature reviews and our careful observations of daily life behaviors. At the same item, its rationality and efficiency must be verified by further empirical researches, which is also one of the most important goals of our future researches.

Of course, there are also a couple of modesty measures that have been developed using Eastern samples. For example, based on survey data gathered from 328 top management team members and 645 middle managers in China, CEO Humility Scale (Ou et al., 2014) was developed. It contains six subscales: transcendent self-concept, self-awareness, openness to feedback, appreciation of others, low self-focus, and self-transcend pursuit. A closer look at the CEO Humility scale reveals that it focuses only on the external behaviors of modest individuals rather than on the intrinsic motivation. Afterward, Hu and Huang (2009) innovatively discussed modesty from the perspective of motivation and developed the Undergraduates’ Self-Modesty Identification Scale (USIS). They argued that motivations underlying individuals’ modest behaviors can be divided into three types: defensiveness, ego integrity and image promotion, and its internal consistency reliability was 0.80. Hu and Huang’s work is truly groundbreaking, but the motivations for modesty they had summarized were somewhat narrow. Therefore, this study tries to explore motivations underlying Chinese people’s modest behaviors deeply and comprehensively. At the same time, it is important to note that these scales focus on only undergraduates; thus, the extent to which the results of these studies generalize to other age groups is unclear.

Based on the classic human values theory (Rokeach, 1973) and the dualistic model of harmony (Leung et al., 2002), Wang et al. (2016) proposed the dualistic model of modesty. According to Wang’s model, self-modesty, also called modesty, refers to a low-key or self-deprecating form of self-presentation one adopts when getting along with others. There are two kinds of modesty among the Chinese: value modesty and instrumental modesty. Value modesty refers to recognizing and persisting in the low-key way of doing things, and graciously accepting the sacrifices modesty may require. People with value modesty believe modesty is a virtue, emphasize that modesty in and of itself is valuable, and firmly regard modesty as the ultimate goal. By contrast, instrumental modesty stresses that the aim of people’s modest behaviors is to realize firmness-restraining flexibility and strength-defeating weakness through unassuming self-presentation (Hu, 2007). People with instrumental modesty stress only modesty’s instrumental value and the main aim of their modest behaviors is to realize other utilitarian purposes. The former is pan-situation and non-utilitarian, and the latter is situated and utilitarian.

**FIGURE 1:**
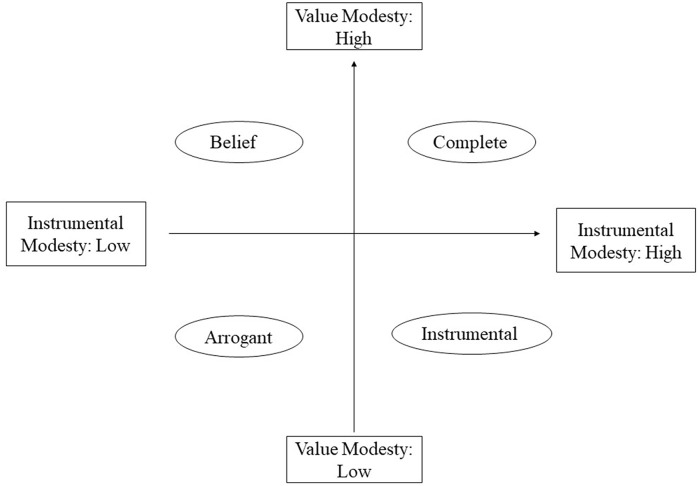
The dualistic model of value and instrumental modesty (Wang et al., 2016).

If we regard value modesty and instrumental modesty as separate dimensions, we could conceptualize a dualistic model of value and instrumental modesty (see Figure 1). As shown in the Figure 1, for Chinese people there are four types of modesty. The first is the *complete type*, which refers to a high emphasis on both value modesty and instrumental modesty. For “complete” types of people, modesty is both a practical means and an ultimate goal. The second is the *belief type*, which refers to a high emphasis on value modesty and a low emphasis on instrumental modesty. People who belong to the “belief” type sincerely take “be modest” as their life motto. The third is the *arrogant type*, which refers to a low emphasis on both instrumental modesty and value modesty. People who belong to the arrogant type do not realize that there is always someone better than they are. The last is the *instrumental type*, which refers to a high emphasis on instrumental modesty and a low emphasis on value modesty. People who belong to this type always regard modesty as an effective means of achieving other utilitarian goals instead of being concerned with its intrinsic values (Wang et al., 2016).

We found that these classical modesty theories mentioned above were compatible with our dualistic model of modesty. First of all, think about the Western models. Tangney (2000) appears to focus more on modesty’s intrinsic values and can apparently subsumed under our value modesty perspectives. Another example is that a number of researchers appeared to be more concerned with modesty’s extrinsic importance in their theories (Fu et al., 2011; Heyman et al., 2011; Han, 2012; Diekmann et al., 2015), which can be subsumed under our instrumental perspectives. For example, Chen et al. (2009) conceptualized modesty as a kind of impression management tactic. Next let’s turn to indigenous models. In classical Chinese philosophy, modesty has always been regarded as a kind of virtue (Li, 1986; Keown, 1992; Chen et al., 2009), which can be subsumed under our value modesty perspective. Hu’s model (2007) is most similar to our dualistic model for both intrinsic and instrumental features of modesty were discussed in these two models. In Hu’s model, Chinese modesty was divided into two distinct types: real modesty and false modesty, and more emphasis was placed on the latter one which can be easily figured out from his definition of modesty: a harmony-control process through unassuming self-presentation, firmness-restraining flexibility, and strength-defeating weakness. It is not hard to see that Hu’s real modesty is similar to our conceptions of Value Modesty, and his false modesty is relevant to our Instrumental Modesty. However, there were also several noteworthy differences between these two models. One major difference is that we portrayed modesty as a value-neutral concept which can be divided into two types (VM and IM) based on its underlying motivations. On the contrary, as discussed above, Hu was more inclined to consider modesty as a means of achieving other utilitarian purposes. Another difference is that, unlike Hu, we considered that these two dimensions of modesty (VM and IM) are not independent, but rather interrelated, which can coexist in a single person. Based on this, we then examine the interplay of value modesty and instrumental modesty to get four modesty styles of Chinese people (see Figure 1). To conclude, the dualistic model of modesty is not a mechanical repetition of Hu’s model, but rather a further expansion. In a word, comparing with these existing models, the dualistic model of modesty can be used as a more coherent way to crystallize the conception of modesty in China and it has been supported by empirical study (Xu, 2016).

Based on the dualistic model of modesty, this study aimed to develop a Chinese Modesty Scale (CMS), which can fit with Chinese culture and be applied to different demographic groups (undergraduates, postgraduates and working people). Based on the results of previous studies demonstrating that personality traits and values continue to change throughout the life span (Leikas and Salmela-Aro, 2015; Shou et al., 2017), we hypothesized that there would be significant differences in both value modesty and instrumental modesty scores among the three groups.

## Materials and Methods

### Item Generation

Based on the dualistic model of modesty, related modesty scales and aforementioned literatures, an in-depth interview outline was formed. The questions included: (1) please talk about your understanding of modesty; (2) what do you think are the motivations for Chinese modesty behaviors? (3) Are there any benefits or disadvantages to being a modest person? A convenience sample of 13 participants knowledgeable about modesty topic was used for the individual interview (four undergraduates, six postgraduates, and three working people; five males and eight females; *M*_age_ = 27.23, *SD* = 3.34); These 10 students were recruited form Nanjing Normal University, and three working people with three or more years of experience are acquaintances of the first and second authors. Each of these 13 interviewees was invited by e-mail to schedule a face-to-face interview which lasted around 1 h. The gender and types composition of these 13 participants is similar to that of all samples used in the formal survey (*N* = 1068). To learn more people’s views of modesty, we then administered an open-ended questionnaire that included similar questions to the in-depth interview. Another 40 participants (20 undergraduates, 10 postgraduates, and 10 working people; 15 males and 25 females; *M*_age_ = 25.17, *SD* = 3.26) were recruited for the open-ended questionnaire. These 30 students were recruited from Nanjing Normal University, and 10 working people are acquaintances of the first and the second authors. Undergraduates and postgraduates who participated in the in-depth interview and open-ended questionnaires in return for 5 yuan (almost 0.78 dollar). The open-ended questionnaire was written in Chinese.

We obtained 44 items through literature analyses, in-depth interviews, and open-ended investigations, and these items were further refined: (1) deleting inappropriate items: ambiguous items and items with high face validity were removed; (2) categorizing: items with similar content were classified as a category; for example, making a favorable impression on others, improving reputation and maintaining a positive social image were grouped into the “image management” category; thus, these 44 items were merged into 20 categories per item. These items were later piloted with 10 psychologists who are familiar with Chinese cultural psychology and modesty, and 20 non-psychologists from Nanjing Normal University, who were asked to rate the clarity and readability of each item, to rate how well each item appeared to measure modesty, to suggest revisions, and to provide additional suggestions. Based on their feedback, the initial scale with 14 items was finally formed. Items were rated on a 5-point Likert scale ranging from 1 (*definitely disagree*) to 5 (*definitely agree*).

**FIGURE 2:**
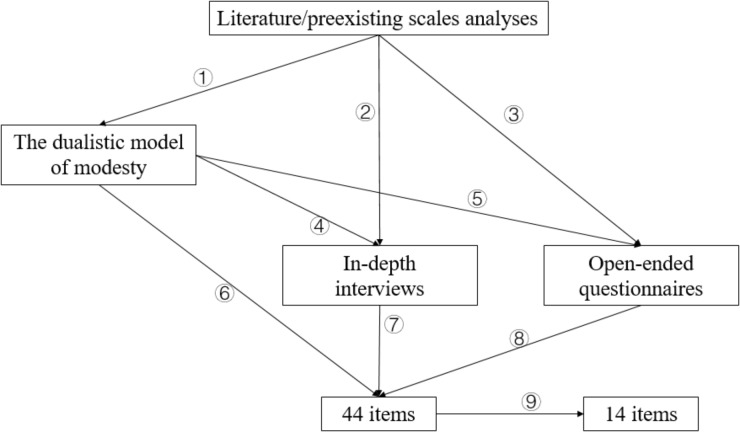
Process of generating initial 14 items of CMS. ①: proposed the dualistic model of modesty based on literature/preexisting scales analyses; ② and ④: formed in-depth interview outlines based on literature/preexisting scales analyses and the dualistic model respectively; ③ and ⑤: devised open-ended questionnaires based on literature/preexisting scales and the dualistic model, respectively; ⑥, ⑦, and ⑧: generated items based on the dualistic model, the results of in-depth interviews and open-ended questionnaire, respectively; ⑨: refined these 44 items to get the initial scale (14 items).

The process of generating items of CMS was presented in Figure 2. As shown, the importance of preexisting scales cannot be ignored. Therefore, to reflect the significance of existing questionnaires and their close relationships with CMS, we presented literature source of each item (see Table 1). When drawn items from existing questionnaires, we comprehensively considered their expressions, psychometric properties and similarity to the objective of this paper. These selected items were then translated, repeatedly reworded, and modified to better reflect the constructs of interest.

**Table 1:** Listing of CMS items and sources.

CMS item	Items adapted from	Reference
1. Modesty can be seen as a traditional Chinese virtue that should be inherited.	Written by first author	
2. I show a modesty behavior in order to make a good impression.^∗^	USIS#6	Hu and Huang, 2009
3. I appreciate modest people, and am happy to interact with them.	HS#28	Elliott, 2010
4. Being modest is helpful to one’s career development.^∗^	CSMQ#24	Liang, 2011
5. Being modest helps me achieve a sense of spiritual well-being.	HS#35	Elliott, 2010
6. Even though modesty sometimes makes me less conspicuous, I still value it as a personality trait.	MRS#12	Whetstone et al., 1992
7. I think downplaying one’s talents and qualities and behaving modestly can be seen as a personal philosophy.	USIS#15	Hu and Huang, 2009
8. Even though behaving modesty may cause me personal losses, I still want to be a modest person.	Written by first author	
9. We should always be modest in our interactions with others, as behind an able person there are always other able people.	MRS#18	Whetstone et al., 1992
10. Modesty is one of the self-cultivation goals that we should pursue.	USIS#12	Hu and Huang, 2009
11. If modesty makes me more prone to misunderstandings, I will no longer behave modestly.	Written by first author	
12. Rather than being modest, we should try to seek opportunities to display our talents.	MRS#4	Whetstone et al., 1992
13. I only behave modesty if it does not have significant negative outcomes for me.	MRS#10	Whetstone et al., 1992
14. If behaving modesty makes others think I am hypocritical, I will no longer behave modestly.	Written by first author	


*USIS to the Undergraduates’ Self-Modesty Identification Scale; HS to the Humility Scale; CSMQ to the College Students Modesty Questionnaire; MRS to the Modesty Responding Scale. ^∗^Means this item was later discarded in EFA and not included in CMS.*

### Participants

The sample comprised 1085 Chinese participants, including undergraduates, postgraduates, and working people. Among them, undergraduates and postgraduates were recruited from Nanjing Normal University, Nanjing Agricultural University, and Hefei Normal University, and working people were recruited through snowball sampling. Participants were excluded for providing obviously repetitive answers (answer 5 on all questions; *n* = 7), or for having missing data in their responses (*n* = 10); therefore, the final sample consisted of 1068 participants (405 undergraduates, 355 postgraduates, and 308 working people; 468 males and 600 females). Two independent samples (sample 1 and sample 2) were randomly generated based on balance of gender and types of participants. We utilized sample 1 (148 undergraduates, 153 postgraduates, and 105 working people; 185 males and 221 females) in the exploratory factor analysis (EFA) and sample 2 (257 undergraduates, 202 postgraduates, and 203 working people; 283 males and 379 females) in the confirmatory factor analysis (CFA), reliability test, and validity test. Almost 67% of participants (*n* = 718) responded to this scale online, while the remaining 33% (*n* = 350) completed paper and pencil measures in the presence of researchers. Participants’ consent was obtained prior to participation, and participants were assured of confidentiality and that that they could terminate their participation at any stage. To obtain test–retest reliability, we asked 40 participants to respond to the same scale 4 weeks later. Demographic information for each sample is shown in Table 2. All 1068 participants are Chinese nationals and were not paid.

**Table 2:** Demographic information for all samples (*N* = 1068).

	Sample 1		Sample 2	
	*N* (%)	*M*_age_ (*SD*)	*N* (%)	*M*_age_ (*SD*)
Gender				
Male	185 (45.60)	27.23 (9.13)	283 (42.70)	24.01 (5.89)
Female	221 (54.40)	26.01 (8.00)	379 (57.30)	27.15 (7.55)
Type of participant				
Undergraduate	148 (36.50)	21.10 (3.82)	257 (38.80)	20.70 (2.01)
Male	71 (47.97)	21.15 (2.89)	163 (63.42)	20.80 (2.20)
Female	77 (52.03)	21.10 (4.53)	94 (36.58)	20.65 (1.66)
Postgraduate	153 (37.70)	24.70 (3.28)	202 (30.50)	24.30 (2.49)
Male	58 (37.91)	25.95 (4.69)	82 (40.59)	24.84 (3.54)
Female	95 (62.09)	23.93 (1.57)	120 (59.41)	24.01 (1.81)
Working people	105 (25.90)	37.00 (9.55)	203 (30.70)	33.80 (7.30)
Male	56 (53.33)	36.27 (10.63)	115 (56.65)	32.14 (6.88)
Female	49 (46.67)	37.78 (8.18)	88 (43.35)	34.41 (7.38)


### Measures

#### Chinese Modesty Scale (CMS)

The 14-item CMS, developed by ourselves, was used. Participants were asked to indicate their level of agreement with each statement using a 5-point Likert scale ranging from 1 (*definitely disagree*) to 5 (*definitely agree*). CMS was written in Chinese.

#### Undergraduates’ Self-Modesty Identification Scale (USIS)

Developed by Hu and Huang (2009), the 15-item USIS was mainly used to measure the implicit structure of Chinese undergraduates’ self-modesty identity commitment. Specifically, its aim is to examine the extent to which individuals identify with modesty’s three functions: defensiveness (avoiding conflicts and achieving interpersonal harmony), ego integrity (constantly urging people to improve their moral cultivation), and image promotion (obtaining favors and positive evaluations from others). Items were rated on a 5-point Likert scale ranging from 1 (*definitely disagree*) to 5 (*definitely agree*). Internal consistency within the current sample was 0.78. Several studies have supported the validity and value of USIS (Huang and Yin, 2012; Wu et al., 2012).

### Procedure

All participants were invited to complete measures in the following order: (1) demographic information; (2) CMS; and (3) USIS.

### Statistical Analysis

Correlation was used for item analysis, items with low item–dimension correlation (*r* < 0.40) were deleted as they may not have a close relevance to the overarching construct of its dimension (Wu, 2013).

An EFA with principal components analysis and a promax oblique rotation were used in sample 1 to examine the structure of the CMS. Bartlett’s test of sphericity and the Kaiser-Meyer-Olkin measure of sampling adequacy (KMO MSA) were used to assess factorability. Items were considered appropriate for factor analysis when Bartlett’s test was statistically significant and the KMO MSA value was 0.80 or higher (Kaiser, 1974). We considered both the Kaiser’s criterion (retaining factors with eigenvalues greater than 1) and scree plot in determining the number of factors to extract. In order to eliminate cross-loadings and aid interpretation of factors, only items that loaded at ≥0.50 were retained, and cross-items were defined as having a secondary factor loading of 0.30 or higher (Bosworth et al., 1999). Factorial simplicity was evaluated by means of the following: (1) the index of factorial simplicity (IFS), with a value ≥0.90 considered meritorious (Kaiser, 1974) and (2) the scale fit index (SFI), with values of at least 0.80 considered desirable (Fleming, 2003). The EFA was conducted using SPSS 21.0.

For the CFA, in this study, the absolute value of skewness coefficients of each item were between 0.01 and 1.75 (<2) and the absolute value of kurtosis coefficients were between 0.49 and 3.35 (<7) (see Table 6); Hence, maximum likelihood (ML) estimation was robust and suitable for estimating the parameters (West et al., 1995; Finney and DiStefano, 2006). However, to increase the credibility and persuasiveness of results, in this study, robust maximum likelihood (robust ML) was used to verify the factor structure previously identified in EFA (Li, 2015). The model fit was evaluated using the normed χ^2^, with a value of <2 considered “very good” (Schreiber et al., 2006) and 2–5 considered “acceptable” (Wu, 2013). We also calculated the standardized root mean square residual (SRMR), with a value of ≤0.08 considered a “good fit” (Hu and Bentler, 1999); the root mean square error of approximation (RMSEA), with a value of ≤0.05 considered a “good fit” and 0.05 to 0.08 a “reasonable fit” (MacCallum et al., 1996); the comparative fit index (CFI), with ≥0.90 considered “good” (Hu and Bentler, 1999); the Tucker Lewis Index (TLI), with a value of ≥0.90 considered great. Given the significant correlation between VM and IM (*r* = -0.09, *p* = 0.02), we also examined the structure coefficients using data from both the pattern coefficients and the factor correlations (Thompson, 1997; Graham et al., 2003; Davis and Finney, 2006). To test the model fit across gender and different types of participants, three levels of measurement invariance analyses, configural invariance, metric invariance, and scalar invariance, were tested. The CFI and RMSEA change values ≤0.01 considered acceptable (Cheung and Rensvold, 2002). The CFA was conducted using Mplus7.

As a more sensible index of internal consistency, Coefficient Omega (OmegaS), instead of Coefficient Alpha, was calculated to estimative consistency reliability using the MBESS package (Dunn et al., 2014; Kelley and Lai, 2012). OmegaS values should exceed 0.50 and 0.75 would be much preferred (Reise et al., 2013). The 4-week test–retest reliability and construct validity of CMS were examined *via* two-tailed Pearson correlations. A subgroup of 40 participants (20 undergraduates, 12 postgraduates, and eight working people; 20 males and 20 females; *M*_age_ = 21.78, *SD* = 3.99) was asked to respond to the same scale 4 weeks later. Average variance extracted (AVE) was used to evaluate CMS’s construct validity, with a value of ≥0.50 considered “accepted” (Fornell and Larcker, 1981). We used USIS to calculate the convergent validity. On the basis of the previous literature, the value modesty subscale was hypothesized to have medium to large positive associations with the ego-integrity factor, whereas the instrumental modesty was hypothesized to have medium to large positive associations with defensiveness and image promotion factors. These analyses were conducted using SPSS Statistics version 21.0.

## Results

### Exploratory Factor Analysis of the CMS

Item 4 was deleted due to its item–dimension correlation being lower than 0.40. The first EFA with a refined 13-item scale was conducted on 202 participants from sample 1. Bartlett’s test of sphericity was significant (*p* < 0.001), and the Kaiser–Meyer–Olkin value was 0.88, indicating that these CMS items were appropriate for factor analysis. Both the eigenvalues and scree plot suggested a two-factor solution, which explained 55.72% of the variance. In the first EFA, Item 2 was deleted as it had a cross-loading problem: its loading in factor 1 was 0.46 and in factor 2 it was 0.36; the other 12 items were retained.

In order to cross-validate the two-factor structure obtained in the first EFA, the second EFA with a refined 12-item scale was then conducted using the remaining 204 participants from sample 1. Bartlett’s test of sphericity was significant (*p* < 0.001), the Kaiser–Meyer–Olkin value was 0.83, and the factor structure obtained through the second EFA was identical to that of the first EFA. All items exhibited strong loadings onto their primary factor without cross-loading and each factor was clearly interpretable (see Table 3). Two factors accounted for 50.75% of the total variance (VM: 34.42%, IM: 16.33%). As for factorial simplicity (shown in Table 3), all individual IFS values (except for item 11; IFS_11_ = 0.446) were above 0.90 and the SFI values were desirable for both the two factors (SFI_1_ = 0.99; SFI_2_ = 0.93).

**Table 3:** CMS second exploratory factor analysis (*N* = 204).

Item	*M* (*SD*)	VM	IM	*h*^2^	IFS
Item 1	4.50 (0.78)	0.74		0.57	0.946
Item 2	4.50 (0.77)	0.80		0.64	0.995
Item 3	4.30 (0.86)	0.82		0.67	0.994
Item 4	4.25 (0.90)	0.79		0.63	0.999
Item 6	4.10 (1.01)	0.63		0.52	0.999
Item 8	3.61 (1.13)	0.52		0.40	0.913
Item 9	4.45 (0.73)	0.66		0.48	0.991
Item 11	4.29 (0.85)	0.74		0.38	0.446
Item 5	3.21 (2.02)		0.72	0.45	0.911
Item 7	3.27 (1.06)		0.69	0.55	0.988
Item 10	2.99 (1.27)		0.74	0.55	0.992
Item 12	3.05 (1.35)		0.65	0.43	0.988
Dimension total		33.99	12.52		
Dimension average		4.25 (=33.99/8)	3.13 (=12.52/4)		
Eigenvalues		4.13	1.96		
Eigenvalues average		0.52 (=4.13/8)	0.49 (=1.96/4)		
Percent variance explained (%)		34.42	16.33		
SFI		0.99	0.93		
OmegaS (CI 95%)		0.86 [0.80, 0.90]	0.67 [0.57, 0.74]		
AVE		0.52	0.49		


*VM, value modesty; IM, instrumental modesty; IFS, index of factorial simplicity; SFI, scale fit index; OmegaS, the Omega coefficients for a subscale; AVE, average variance extracted.*

The results of the EFA were consistent with the original theoretical model. Based on this model, we named the first eight-item factor “value modesty” (i.e., modesty is a kind of Chinese traditional virtue that we should inherit), and named the second four-item factor “instrumental modesty” (i.e., only when modesty does not allow me to make substantial losses can I be modest).

### Confirmatory Factor Analysis of the CMS

**FIGURE 3:**
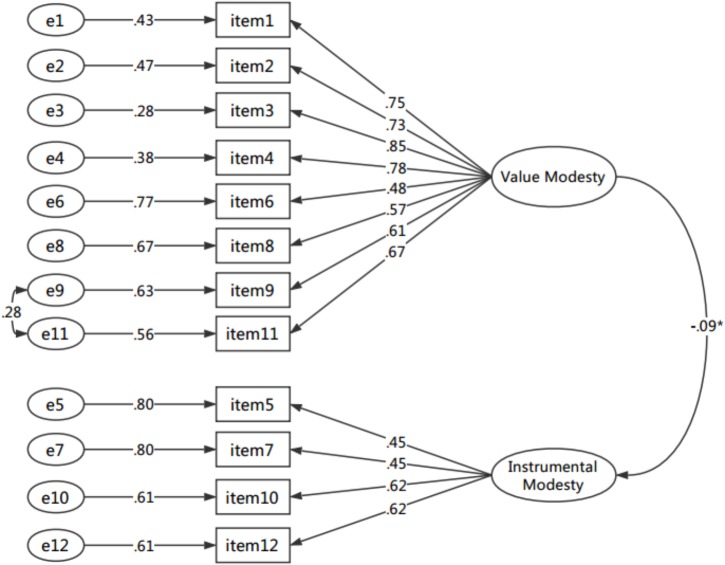
CFA results on CMS scale.

The CFA was conducted in order to replicate the factor structure identified *via* the EFA with a new and independent sample of participants (*n* = 662). This study tested two models for the CMS: a one-factor model and a two-factor model. The former refers to a simple primary model that all 12 items were affected by the same latent variable, and the two-factor model was developed based on the EFA results. In the two-factor model, modification indices (MI) indicated that a path need to be added between item 9 and item 11. This suggestion is deemed reasonable given that item 9 (“We should always be modesty in our interactions with others, as behind an able person there are always other able people”) and item 11 (“Modesty is one of the self-cultivation goals that we should pursue”) belong to the same dimension VM, and these two items are both concerned with recognizing and persisting the low-key way of behaving and holding that modesty is a virtue. Freeing the parameter between items 9 and 11 substantially improved the fitness of two-factor model: normed χ^2^ = 3.40, RMSEA = 0.06, SRMR = 0.04, CFI = 0.95, and TLI = 0.94. The goodness-of-fit indices of each model (see Table 4) show that compared with the one-factor model, the two-factor model fitted better; consequently, the latter was accepted as the final model. The results of CFA for two-factor model are illustrated in Figure 3. All standardized factor loadings were statistically significant (*p* < 0.001), and all items significantly loaded onto the same factor in the CFA as they had in the EFA. At the same time, as shown in Table 5, factor pattern and structure coefficients indicated that all the items correlated higher with their corresponding latent factor.

**Table 4:** Comparison of fitting indices of models (*N* = 662).

Model	χ^2^	*df*	Normed χ^2^	RMSEA	SRMR	CFI	TLI
One-factor	636.10	79	8.05^∗∗∗^	0.10	0.09	0.80	0.77
Two-factor	176.76	52	3.40^∗∗∗^	0.06	0.04	0.95	0.94


*^∗^p < 0.05, ^∗∗^*p* < 0.01, ^∗∗∗^*p* < 0.001.*

**Table 5:** Factor pattern and structure coefficients for the CMS (*N* = 662).

Item	VM		IM	
	Pattern	Structure	Pattern	Structure
Item 1	0.75	0.75	0	–0.07
Item 2	0.73	0.73	0	–0.07
Item 3	0.85	0.85	0	–0.08
Item 4	0.78	0.78	0	–0.08
Item 6	0.48	0.48	0	–0.05
Item 8	0.57	0.57	0	–0.06
Item 9	0.61	0.61	0	–0.06
Item 11	0.67	0.67	0	–0.07
Item 5	0	–0.04	0.45	0.45
Item 7	0	–0.04	0.45	0.45
Item 10	0	–0.06	0.62	0.62
Item 12	0	–0.06	0.62	0.62


In terms of measurement invariance across gender, two-group CFAs were conducted to test for the comparability of the CMS between men and women. The data indicated configural invariance (CFI = 0.934, TLI = 0.917, RMSEA = 0.066, and SRMR = 0.049) across gender was supported. The CFI difference test (ΔCFI) between the configural and metric invariance model was 0.002 (≤0.01) and the RMSEA difference (ΔRMSEA) was -0.004 (≤0.01), which indicated metric invariance across gender was supported. Change values between the metric and scalar invariance (ΔCFI = 0.004, ΔRMSEA < 0.001) demonstrated that full scalar invariance was supported. These findings indicated that the structure of CMS does measure the same construct in Chinese men and women.

Three-group CFAs were conducted for the comparability of the CMS among different types of participants (undergraduates, postgraduates and working people). The data indicated configural invariance (CFI = 0.943, TLI = 0.928, RMSEA = 0.061, and SRMR = 0.053) was supported. Change values between the configural and metric invariance model (ΔCFI = 0.002 and ΔRMSEA = 0.005) demonstrated that metric invariance was supported. Change values between the metric and scalar invariance (ΔCFI = 0.022 and ΔRMSEA = 0.007) demonstrated that scalar invariance was not supported (ΔCFI > 0.01). Partial scalar invariance was then conducted and an ideal model was retained (ΔCFI = 0.010 and ΔRMSEA = 0.006) after relaxing the constrains on the intercepts of item 7. The fit of all the models was shown in Table 7.

### Psychometric Properties of the CMS

The item correlations were acceptable (see Table 6) and the internal consistency reliability (OmegaS) was high (VM: 0.86; IM: 0.67) (see Table 3). The 4-week test–retest reliability of CMS was great (VM: *r* = 0.90, *p* < 0.001; IM: *r* = 0.86, *p* < 0.001).

**Table 6:** CMS descriptive and correlation information (*N* = 662).

Item	Skew	Kurtosis	1	2	3	4	6	8	9	11	5	7	10	12
VM			0.75**	0.73**	0.81**	0.80**	0.63**	0.69**	0.70**	0.75**				
Item 1	–1.75	3.35	1											
Item 2	–1.71	3.11	0.57**	1										
Item 3	–1.18	1.17	0.64**	0.66**	1									
Item 4	–1.13	0.92	0.59**	0.54**	0.68**	1								
Item 6	–0.95	0.22	0.36**	0.31**	0.35**	0.39**	1							
Item 8	–0.36	–0.72	0.38**	0.34**	0.45**	0.50**	0.37**	1						
Item 9	–1.46	2.82	0.45**	0.50**	0.49**	0.45**	0.40**	0.38**	1					
Item 11	–1.25	1.62	0.51**	0.46**	0.55**	0.51**	0.41**	0.44**	0.57**	1				
IM											0.76^∗∗^	0.55^∗∗^	0.68^∗∗^	0.70^∗∗^
Item 5	1.73	2.27	–0.09*	–0.06	–0.09*	–0.09*	0.06	–0.16**	–0.03	–0.05	1			
Item 7	–0.09	–0.49	–0.05	0.01	0.02	–0.06	–0.04	–0.04	0.03	0.01	0.16^∗∗^	1		
Item 10	0.01	–1.03	–0.02	0.05	0.02	–0.01	0.06	–0.08*	0.03	–0.01	0.28^∗∗^	0.32^∗∗^	1	
Item 12	–0.03	–1.16	–0.08*	–0.10**	–0.07	–0.06	–0.05	–0.12**	–0.09	–0.11	0.31^∗∗^	0.27^∗∗^	0.37^∗∗^	1


*^∗^p < 0.05, ^∗∗^*p* < 0.01, ^∗∗∗^*p* < 0.001.*

**Table 7:** Analysis of measurement invariance across gender and type of participants (*N* = 662).

Model	S-B*χ*^2^	*Df*	CFI	TLI	RMSEA	SRMR		ΔCFI	ΔRMSEA
Measurement invariance across gender				
M1: configural invariance	281.30***	104	0.934	0.917	0.066	0.049			
M2: metric invariance	298.44***	114	0.936	0.926	0.062	0.055	M2:M1	0.002	–0.004
M3: scalar invariance	317.63***	124	0.932	0.928	0.062	0.057	M3:M2	0.004	0.000
Measurement invariance across type of participant				
M1: configural invariance	316.64***	156	0.943	0.928	0.061	0.053			
M2: metric invariance	343.27***	176	0.945	0.938	0.056	0.060	M2:M1	0.002	0.005
M3: scalar invariance	418.19***	196	0.923	0.922	0.063	0.067	M3:M2	0.022	0.007
M4: partial scalar invariance	409.12***	194	0.935	0.924	0.062	0.066	M3:M2	0.010	0.006


*^∗^p < 0.05, ^∗∗^*p* < 0.01, ^∗∗∗^*p* < 0.001.*

The structure validity of the scale was analyzed. The results showed that factor total correlations (*r*_vm_ = 0.78, *p* < 0.001; *r*_im_ = 0.56, *p* < 0.001) were higher than the correlation between the value modesty factor and the instrumental modesty factor (*r* = -0.09, *p* = 0.02), indicating that these two factors have a certain degree of independence but are also interrelated, and have great consistency with the overall concept. These results showed that the two-factor theoretical structure of the CMS is strong. In addition, the AVE ranged from 0.49–0.52 (Table 3), which indicated adequate levels of construct validity (Fornell and Larcker, 1981).

We used USIS to calculate the CMS’s convergent validity. As shown in Table 8, the CMS displayed strong convergent validity: the value modesty dimension had the highest degree of correlation with the ego-integrity factor (*r* = 0.60, *p* < 0.001), and the correlation of the instrumental modesty dimension to Hu and Huang’s two factors, defensiveness (*r* = 0.23, *p* < 0.001) and image promotion (*r* = 0.26, *p* < 0.001), was higher than that of the ego-integrity factor (*r* = -0.06, *p* = 0.15).

**Table 8:** Correlation between CMS and USIS.

	VM	IM	Defensiveness	Ego-integrity	Image promotion
VM	–				
IM	–0.09*	–			
Defensiveness	0.53**	0.23**	–		
Ego-integrity	0.60**	–0.06	0.55**	–	
Image promotion	0.39**	0.26**	0.48**	0.49**	–


*VM, value modesty; IM, instrumental modesty. Defensiveness, Ego-integrity, and Image promotion are the three dimensions of the USIS. ^∗^*p* < 0.05, ^∗∗^*p* < 0.01, ^∗∗∗^*p* < 0.001.*

### Relationships of the CMS With Demographic Variables

Some independent-samples *t* tests were conducted to investigate whether people of different genders conceive of modesty differently. The results showed that males (*M* = 13.03, *SD* = 4.23) scored higher than females (*M* = 12.14, *SD* = 3.62) on the instrumental modesty subscale, *t* (660) = 2.92, *p* = 0.004, and there was no significant difference between males and females on the value modesty dimension, *t* (660) = 0.17, *p* = 0.87. In order to investigate the relationship between age, VM, and IM, Pearson correlation tests were computed. Results showed that age was positivity correlated with VM (*r* = 0.21, *p* < 0.001), but not with IM (*r* = -0.07,*p* = 0.06).

We used one-way ANOVA to evaluate differences among three types of participants. In the value modesty dimension, compared with undergraduates (*M* = 33.59, *SD* = 5.13) and postgraduates (*M* = 33.11, *SD* = 5.11), working people scored higher (*M* = 35.37, *SD* = 5.13), *F* (2,661) = 11.48, *p* < 0.001, partial eta squared = 0.03, and there was no significant difference between undergraduates and postgraduates (*p* = 0.94). In the instrumental modesty dimension, there were no statistically significant differences among the three groups, *F* (2,661) = 0.49, *p* = 0.62.

## Discussion

Currently, three methods can be used to construct psychology theory: theory-driven, data-driven, and a combination of theory-driven and data-driven (Tang and Guo, 2015). Psychological theory constructed through the third way will likely produce more robust models and measures than the other two methods (Jiang, 2004). Therefore, the third way was used in this study to gain a clearer picture of Chinese modesty; first, we generated preliminary items based on Wang’s modesty model, and then revised it according to empirical results obtained from a sample of 1068 Chinese.

It must be made clear that, from the beginning, CMS was developed with the purpose of measuring one’s views of modesty rather than his/her levels of modesty. Taking the instruction of CMS as an example, during the test period, each participant was given the same instruction, “This scale is designed to investigate your personal ‘views’ on some issues and there are no right or wrong answers. Please read each item carefully and indicate the extent to which you agree or disagree with each statement.” As shown, it was distinctly presented in the instruction that this scale was designed to measure participants “views” rather than “levels.” Following this original intention of investigation, all items included in CMS are suitable for measuring responses’ views of modesty. For example, “modesty can be seen as a traditional Chinese virtue that should be inherited.” And another example, “I think downplaying one’s talents and qualities and behaving modestly can be seen as a personal philosophy.” However, not all of these 12 statements can be used to measure one’s levels of modesty, just consider again the two examples mentioned above. Also, we have to admit that it is difficult for psychological scales to truly measure respondents’ levels. To be specific, as demonstrated in the systematic distortion hypothesis (Shweder and D’Andrade, 1979), there are significant “deviations” or “distortions” between individual’s responses in psychological questionnaires and his/her actual behaviors. In other words, participant’s scores obtained from self-reported psychological scales are not equal to his/her real behaviors, and these scores just represent one’s views of the given topic. Thus, as a newly developed personality scale, CMS may be unable to measure one’s modesty level but only obtain his/her views of modesty.

### CMS Reliability and Validity

In the item analysis, the original item 4, being modest is helpful to one’s career development was deleted as its item–dimension correlation was lower than 0.40. In this item, 77% of participants selected four points or above, suggesting most participants “mostly agree” or “definitely agree” that modesty has positive effects on one’s career; this view held by most Chinese is consistent with previous empirical results. For instance, Wosinska et al. (1996) found that, comparing with boastful presenters, modest self-presenters were more favored and receive more willing supports from others. Blickle et al. (2012) also argued that, for employees high in political skills, increases modesty is associated with higher subsequent hierarchical position and career satisfaction.

The structure of Chinese modesty obtained in EFA was in line with Wang’s theory. Furthermore, we argued that value modesty is a stronger explanatory factor than instrumental modesty in explaining Chinese modesty based on following reasons: (1) VM has higher eigenvalue and percent variance explained than IM (VM: 4.13, 34.42%; IM: 1.96, 16.33%) and (2) VM has a higher eigenvalue average than IM (0.52 > 0.49). In this study, eigenvalues average was calculated as a supplement to eigenvalues and percent variance explained for these two indices can be greatly affected by the number of items in one factor. In specific, the more items one factor has, the higher eigenvalues average and percent variance explained it may get. In VM factor, the actual variance extracted is 4.13 and the eigenvalues average is 0.52 (4.13/8); in IM factor, the actual variance extracted is 1.96 and the eigenvalues average is 0.49 (1.96/4); (3) the factor total score of VM (33.99) was almost three times higher than that of IM (12.52); (4) the factor average of VM (4.25 = 33.99/8) was much higher than that of IM (3.13 = 12.52/4); and (5) the item average of VM (3.61–4.50) was a lot higher than that of IM (2.99–3.05). All these results indicated that, comparing with IM items, VM items have a higher contribution to the total variances (results 1–2), and participants have a higher agreement with VM items (results 3–5). Hence, we can conclude that modern Chinese people who grew up in an atmosphere of encouraging competition and individuality still think highly of the intrinsic value of modesty and regard it as a traditional inherited virtue. This belief, rather than pursuing other utilitarian ends, tends to drive Chinese people to maintain modesty all the time.

Given coefficient Omega’s advantages over coefficient Alpha (for a more detailed description, see Dunn et al., 2014), it is universally recommended that researchers should change to report coefficient Omega in place of coefficient Alpha (Watkins, 2017). Specifically, coefficient Alpha is rarely appropriate for its strict assumptions. For example, with regard to one of its assumptions – tau-equivalent model, there is a general consensus among researchers that this requirement is hard to reached (Fife et al., 2012; Gignac, 2014). Consequently, in this study, we calculated OmegaS in place of coefficient Alpha for our data did not reach the tau-equivalent assumption. Moreover, coefficient Omega can be calculated along with confidence intervals (CIs) which can provide a more accurate degree of confidence in the scale’s consistency.

Hu and Huang’s USIS was used to evaluate convergent validity. Consistent with expectations, people who scored high in the value modesty dimension found Hu and Huang’s ego-integrity factor more attractive; besides, people who scored high in the instrumental modesty dimension put more emphasis on defensiveness and image promotion factors. Thus, we can conclude that this scale has good convergent validity. It is important to note that this scale is not a mechanical repetition of Hu and Huang’s research, but rather a further expansion of Chinese modesty measurement based on previous studies. In the value modesty dimension, Hu and Huang emphasized only modesty’s ego-integrity function without considering other intrinsic values of modesty, for example, as a kind of traditional virtue, where modesty in and of itself is attractive to Chinese people. In the instrumental modesty dimension, Hu and Huang focused only on modesty’s defensiveness and image promotion function without considering other external motivations, such as avoiding overstating, preventing unnecessary pressures caused by excessive attention and so on.

It must be pointed out that instrumental modesty is a subtype of modesty. In fact, the instrumental importance of modesty has always been acknowledged. For example, a number of researchers appeared to be more concerned with modesty’s extrinsic importance rather than its intrinsic values in their theories (Fu et al., 2011; Heyman et al., 2011; Han, 2012; Diekmann et al., 2015). Some of them equate modesty directly with instrumental modesty. As demonstrated in the Figure 3, the correlation between VM and IM was significant but low (*r* = -0.09, *p* = 0.02). However, we cannot draw a conclusion that these two dimensions don’t belong to the same structure. Try to see this issue from a different perspective, this low but significant result indicates that, in Chinese eyes, VM is not only related but also quite distinct from IM, which was compatible with our dualistic model.

### Modesty and Demographic Variables

The results show that male and female participants differed in their ratings on the instrumental modesty scale. Compared with females, males preferred instrumental modesty. Perhaps the traditional division of family roles contributed to this difference. The idea “men outside, women inside” is deeply rooted among the Chinese. Compared with women, men have greater family pressures and are more eager to achieve success in their careers, so they are more likely to adopt practical strategies in the process of interpersonal interaction; that is, to pay more attention to instrumental modesty.

By and large, participants in previous modesty research can be divided into two categories. First, 7- to 14-year-old children, whose modesty behavior development has been of great interest to researchers (Lee et al., 1997; Fu et al., 2010). Second, undergraduates, whose views of modesty have been examined using numerous self-report measurements. To develop a measure that has a wider application scope and higher practical value, this study investigated 1068 Chinese people aged 20–53, including undergraduates, postgraduates and working people. We found that in the value modesty dimension, working people scored higher than undergraduates and postgraduates, indicating that compared with young students, people with rich life experience put more emphasis on the intrinsic value of modesty. Maybe it is because they are more affected by Chinese traditional culture and less affected by Western culture than students, hence they truly believe that modesty in and of itself is a virtue.

### Limitations and Future Directions

In contrast to previous studies that only focused on undergraduates and developed theories through the data-driven method, this study focused on a broader range of people and was based on a combination of the theory-driven and data-driven methods. Another advantage of the CMS is that it is suited to Chinese culture. However, for a correct interpretation of our results, it is necessary to consider its limitations. First, the lack of elderly participants who were involved in the scale’s development. Obviously, there could be large differences between experienced elderly people and inexperienced young people in their modesty views. This study did not explore elderly people’s modesty due to sampling limitations, which may limit the generalizability of the findings of the current study to all Chinese. Second, self-reported modesty scales have struggled with social desirability (Elliott, 2010). This scale was completed anonymously, and participants were requested to “please answer this scale according to your actual situation.” These procedures, to a certain degree, may reduce participants’ response biases, but still cannot eliminate them. Future researches are expected to figure out whether there are more reasonable and effective ways to measure modesty, and the validity of the CMS need further testing. Third, future researchers should investigate commonalities and differences among individuals with different types of modesty (*complete type*, *belief type*, *arrogant type* and *instrumental type*). For example, explore whether there is significant difference in narcissism between *complete type modesty* people and *arrogant type modesty* people.

## Conclusion

(1) The dualistic model of modesty was confirmed, and the CMS scale duly included two subscales: value modesty and instrumental modesty. (2) As a reliable and valid measure, this scale can be used to measure Chinese modesty in different age groups.

## Ethics Statement

This study was carried out in accordance with the recommendations of professional code of ethics of psychometric workers, Chinese Psychological Society. All subjects gave informed consent in accordance with the Declaration of Helsinki. Participants were allowed to withdraw from the study whenever they wanted and the data were collected anonymously.

## Author Contributions

The contribution of MX was in item generation, data collection, data analysis, and writing the paper. The contribution of FW was in providing theoretical guidance and writing the paper. The contribution of RC was in the item generation and data collection.

## Conflict of Interest Statement

The authors declare that the research was conducted in the absence of any commercial or financial relationships that could be construed as a potential conflict of interest.
